# Congenital Knee Dislocation: A Case Report

**DOI:** 10.7759/cureus.96469

**Published:** 2025-11-10

**Authors:** Anaisa Afonso, Gabriela Botelho, Joana Arcangelo, Joana Soares, Marta Aguiar

**Affiliations:** 1 Pediatrics, Unidade Local de Saúde Arrábida - Centro Hospitalar de Setúbal, Setúbal, PRT; 2 Pediatrics, Unidade Local de Saúde do Alentejo Central, Évora, PRT; 3 Pediatric Orthopedics, Hospital Dona Estefânia, Lisbon, PRT; 4 Neonatology, Hospital de São Francisco Xavier - Unidade Local de Saúde Lisboa Ocidental, Lisbon, PRT; 5 Neonatology, Centro Hospitalar Lisboa Ocidental, Lisbon, PRT

**Keywords:** congenital abnormalities, congenital knee dislocation, developmental dysplasia of the hip, ligamentous laxity, newborn

## Abstract

Congenital knee dislocation (CKD) is a rare musculoskeletal condition that may occur in isolation or be associated with other congenital abnormalities. Prognosis depends on the severity of the deformity and the timing of diagnosis and treatment. Comprehensive assessment and close follow‑up are essential to optimize recovery and to exclude associated anomalies. We report the case of a female newborn with CKD, diagnosed postnatally, associated with developmental dysplasia of the hip (DDH), generalized ligamentous laxity, congenital heart disease, and a chromosomal abnormality. Early conservative management, started in the first hour of life, resulted in successful reduction and complete recovery of knee mobility.

## Introduction

Congenital knee dislocation (CKD), also termed *genu recurvatum congenitum*, is a rare condition characterized by hyperextension of the knee with variable severity [1-4]. The severity grade classification, according to Leveuf and Pais, is based on femorotibial alignment and the degree of passive knee flexion and ranges from mild hyperextension, with passive flexion >90° (Grade I), through subluxation (Grade II), to complete dislocation, in which the tibia lies anteriorly displaced over the femoral condyles, with passive flexion <30° (Grade III) [1,4-7]. Hyperextension up to 20°, with preserved mobility, may be seen in breech neonates or those with a family history of ligamentous laxity, requiring no treatment [8].

The estimated incidence of CKD is one per 100,000 live births, accounting for approximately 1% of cases of developmental dysplasia of the hip (DDH) [1,4,8-11]. It is more common in breech deliveries (21-40% of cases), has female predominance (2:1 ratio), and is bilateral in one‑third of patients [1,3,8,9,12,13]. Most cases are diagnosed at birth, although prenatal diagnosis may occur in about 20% of cases, usually during the second trimester [1,3-5,8,12].

The etiology remains unclear, involving extrinsic (mechanical) and intrinsic factors [3-5,12,14-16]. Implicated mechanical factors include reduced intrauterine space, oligohydramnios, multiple gestation, abnormal fetal positioning, breech presentation, anterior cruciate ligament anomalies, or quadriceps contracture [8,10,12,14]. Intrinsic factors are related to genetic and neuromuscular abnormalities [2]. Familial cases have been reported, but no specific causative gene has yet been identified [3,12,14,15].

CKD may occur alone or in association with other musculoskeletal conditions, most frequently DDH (45-70% of cases) and clubfoot (30-50%). Other associations include elbow dislocation, palate abnormalities, iliotibial band adhesions, patellar dysplasia, and hamstring contractures [2,4,8,9,12-14]. Due to its strong association with DDH, a hip ultrasound is recommended at the time of CKD diagnosis [9]. CKD and DDH share pathophysiological features, such as restricted movement, instability, muscle contracture, and abnormal skin folds, and both conditions occur more frequently in females [17]. In rare cases, CKD may be associated with myelomeningocele or syndromic associations, including arthrogryposis, achondroplasia, osteogenesis imperfecta, Larsen syndrome, Ehlers-Danlos syndrome, Marfan syndrome, and Noonan syndrome [1,4,5,14,16,18].

Joint mobility and the presence of anterior knee skin folds are favorable prognostic indicators, reflecting a shorter intrauterine duration and greater likelihood of successful reduction [9,17]. Conversely, the absence of anterior skin folds may indicate prolonged intrauterine dislocation, often associated with quadriceps tendon shortening, reduced mobility, and increased difficulty in successful reduction [19].

Clinical and anatomical findings may include palpable femoral condyles in the popliteal fossa, a non-palpable or absent patella, external tibial rotation, valgus deformity, quadriceps shortening or fibrosis, tight anterior capsule, suprapatellar pouch hypoplasia, and shortening of the cruciate ligaments [4,5,7,9].

## Case presentation

The patient was the second child of a healthy 40‑year‑old mother, a late preterm baby girl of 35 weeks and three days, and prenatal echocardiography revealed a perimembranous ventricular septal defect and an overriding aorta. Chromosomal microarray analysis by amniocentesis showed a duplication in region 22q11.22-q11.23.

**Figure 1:**
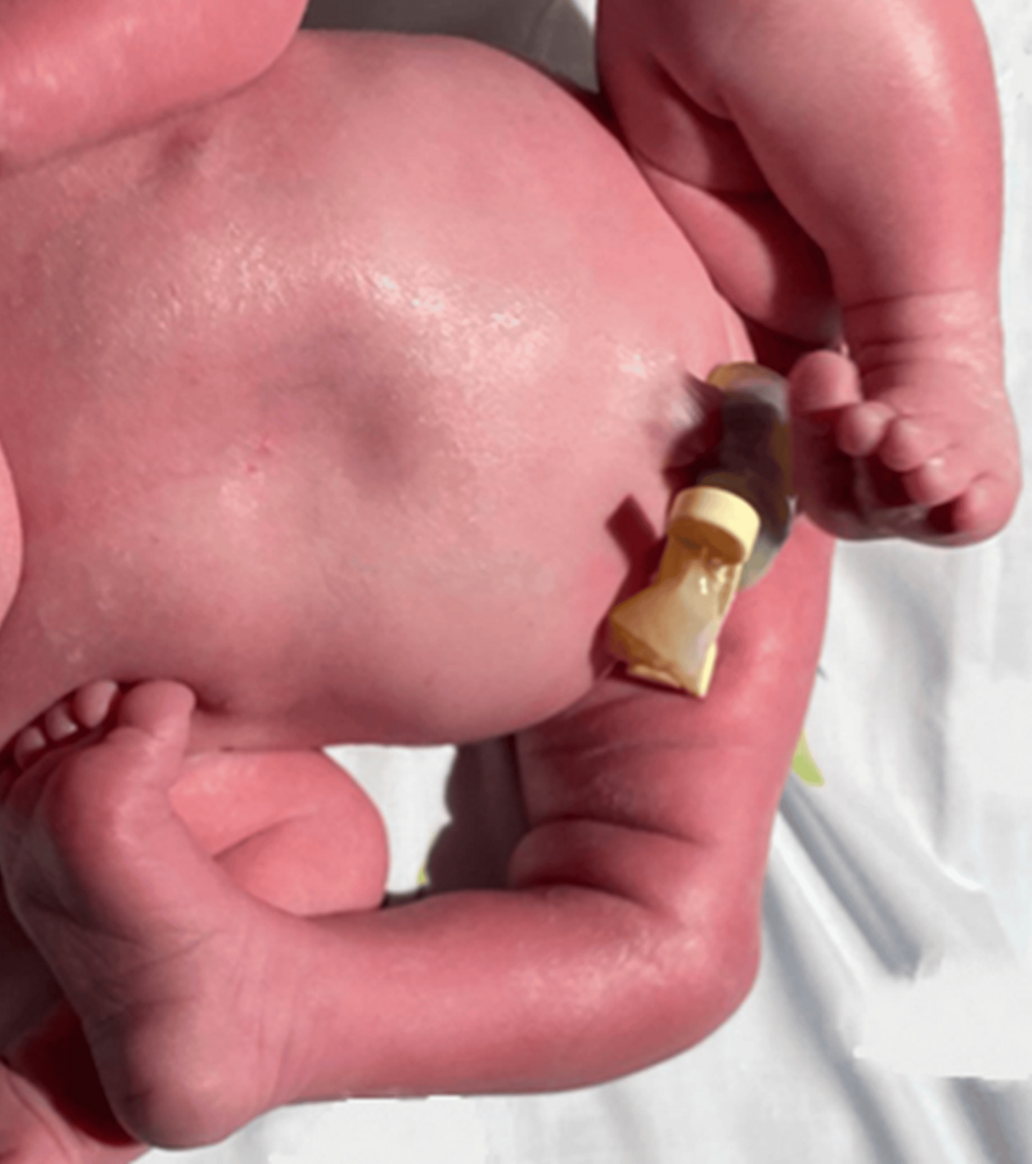
Right CKD at birth, with hyperextension at –90° CKD: congenital knee dislocation

The newborn was delivered vaginally in cephalic presentation. Apgar scores were 9 and 10 at one and five minutes, respectively. Birth weight was between the 50th and 90th percentiles, and length was between the 10th and 50th percentiles. Postnatal examination revealed right CKD, Grade II, with hyperextension at -90°, without vascular compromise (Figure 1).

**Figure 2:**
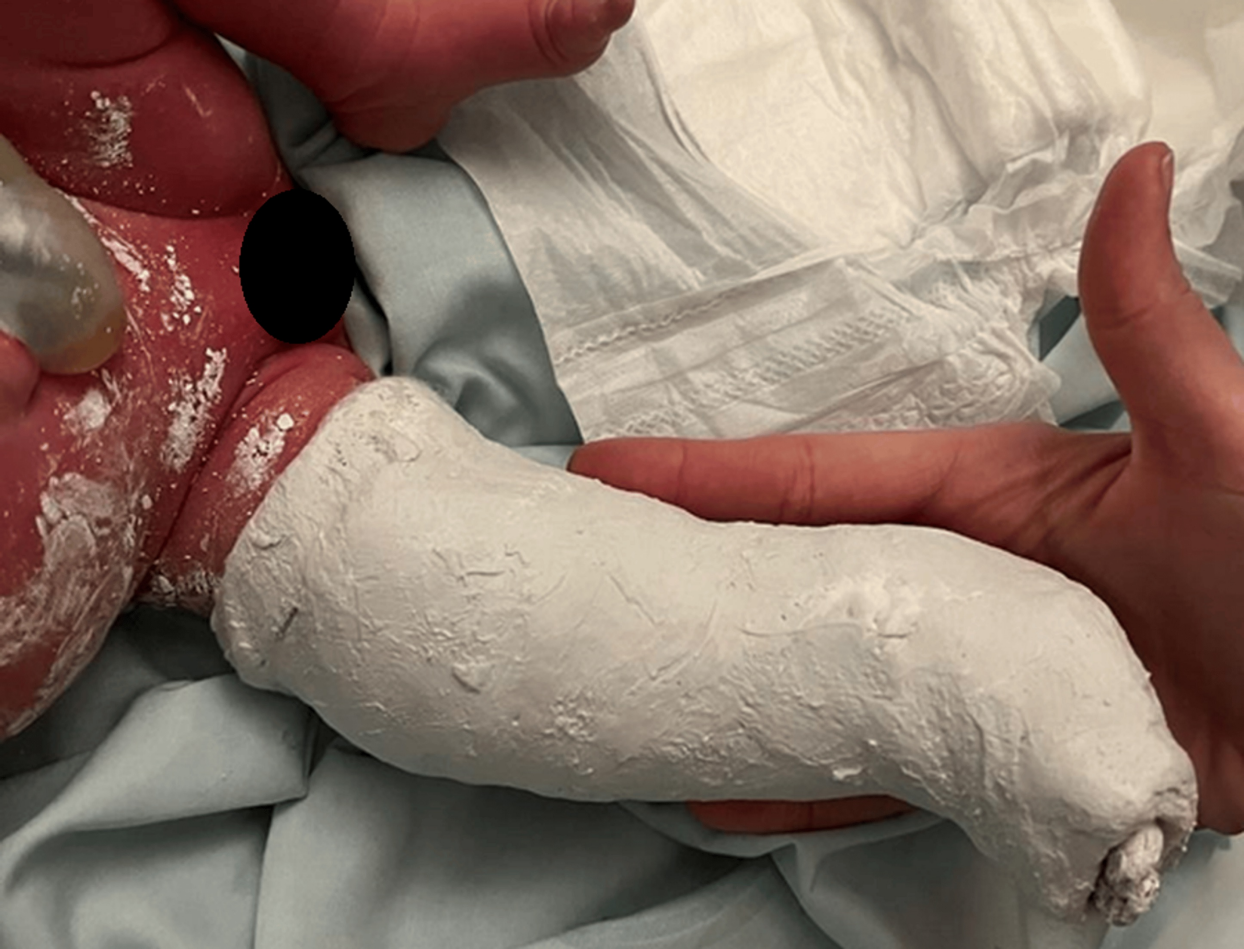
Right lower limb following plaster cast at 5–10°

**Figure 3:**
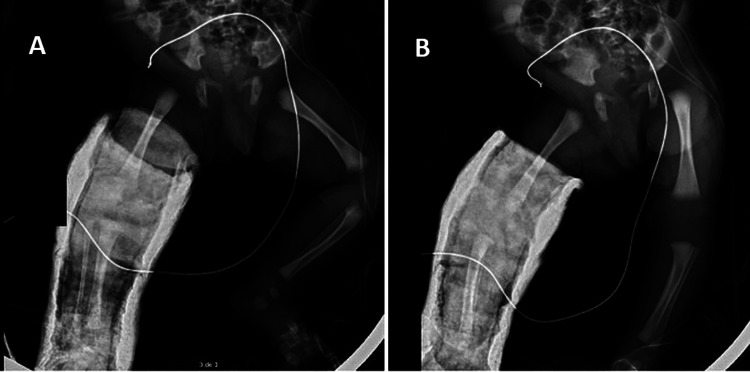
Radiographs of the right lower limb after a plaster cast at 5–10° A: anteroposterior; B: lateral views.

Partial reduction was performed by the orthopedic team, followed by immobilization in a plaster cast at 5-10° flexion (Figure 2), with radiographic confirmation (Figure 3).

The neonate was admitted for five days in the neonatal intensive care unit. Additional findings included a high‑arched palate and congenital heart disease: moderate‑to‑large secundum atrial septal defect, nonrestrictive perimembranous ventricular septal defect with overriding aorta <50%, and a small apical muscular ventricular septal defect.

Passive hyperextension of 10° and passive flexion of 110° were observed on day 8 of life. A new plaster cast was applied at 90° flexion. At 30 days, following cast removal, mobility was preserved, and full painless flexion was achieved. During that evaluation, the Ortolani sign was positive in the right hip. Physiotherapy was initiated, achieving complete recovery of knee motion.

**Figure 4:**
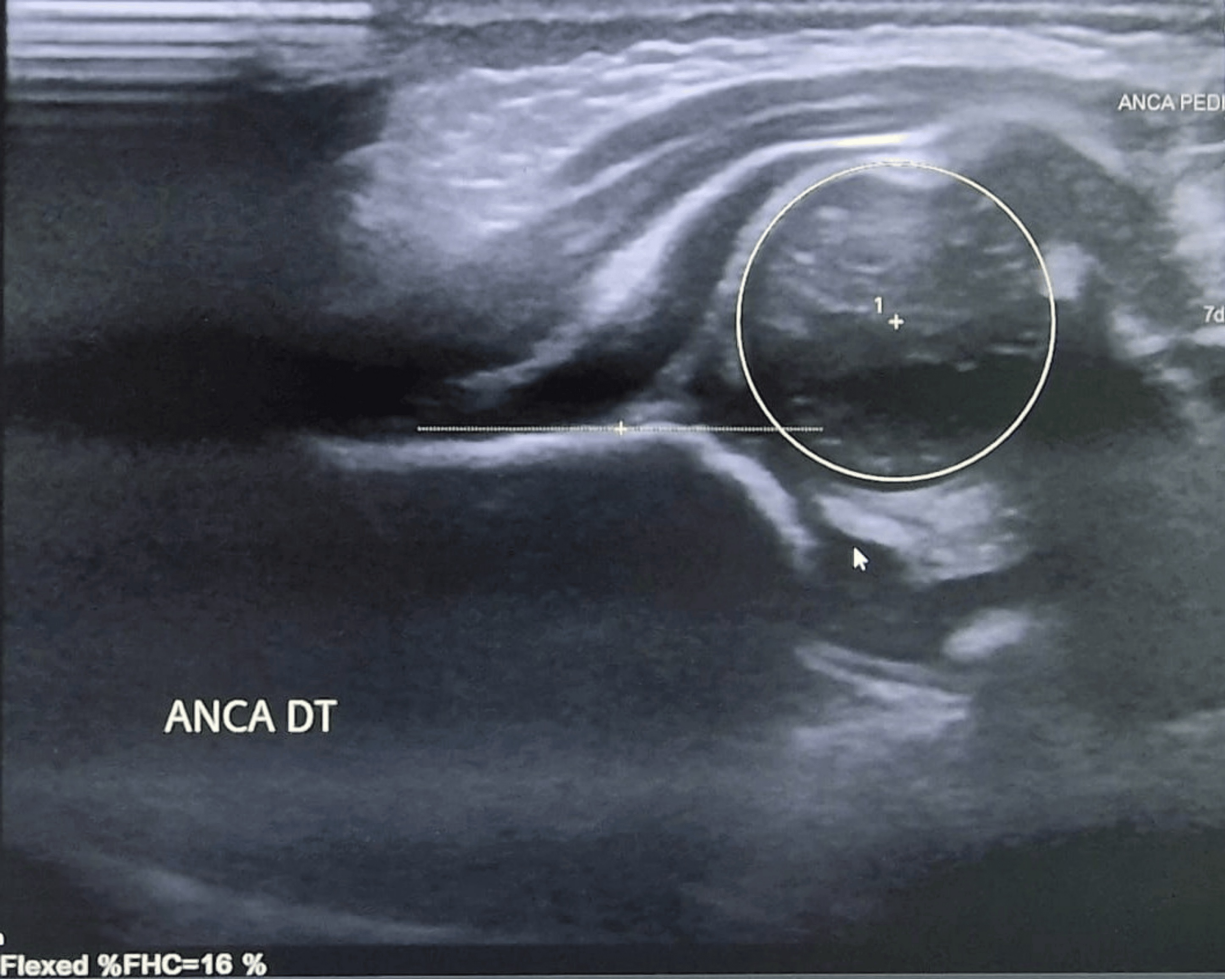
Hip ultrasound at six weeks of age showing right-sided developmental dysplasia of the hip (DDH), with femoral head coverage (FHC) of 16% in flexion

At six weeks, DDH was confirmed on ultrasound (Figure 4), and a Pavlik harness was applied until 4.5 months of age.

Ligamentous hyperlaxity persisted. Additional diagnoses included positional plagiocephaly, managed with physical medicine and rehabilitation treatment, and axial hypotonia was noted at three months. At five months, surgical closure of the ventricular septal defect was performed. The patient has been under multidisciplinary follow-up for growth and neurodevelopment since birth, showing normal motor and global developmental milestones, and achieved independent walking at 15 months of age.

## Discussion

CKD is a rare congenital anomaly. Early recognition and treatment are crucial to restore mobility and prevent long‑term disability [14]. Radiographs in maximum extension and flexion are useful for severity grading, and ultrasound assists in excluding associated abnormalities [17].

Conservative treatment is the first‑line option, consisting of serial casting until 90° flexion is achieved, followed by physiotherapy [2,4,5,13]. Aggressive manipulation should be avoided due to the risk of deformity, fractures, physeal injury, or vascular compromise [7]. Surgery may be required in severe or refractory cases, particularly when treatment is delayed (beyond five weeks, manipulation success is reduced to 27%). Ideally, the treatment should be performed before the child begins standing, but no later than two years of age [4,5,8,9,11,18].

Surgery indications include failure to achieve 30° flexion after three months of casting, particularly in Grade II or III deformities, and recurrent dislocations [9,14]. When other musculoskeletal anomalies coexist, treatment should prioritize correction of CKD before addressing other conditions. Notably, in cases involving both hip and knee dislocations, hip reduction may occur spontaneously after correction of the knee [2,4,8,14].

Prognosis depends on the severity of deformity, range of motion, presence of anterior knee folds, degree of hyperlaxity, comorbidities, and optimal management [3,5]. Early conservative management (ideally within 48 hours of birth) is associated with favorable outcomes. Favorable prognostic factors include joint reducibility, maintained knee stability, and the presence of anterior skin grooves. Conversely, unfavorable outcomes are frequently associated with syndromic presentations, passive knee flexion below 50°, and the absence of anterior skin grooves [9].

Given the frequent association of CKD with other pathologies, careful evaluation for DDH and other osteoarticular disorders is mandatory [2,4,17]. Although long‑term prognosis is usually favorable, structured multidisciplinary follow‑up is needed to optimize recovery and address associated conditions [2,4,5,10,12]. Rare complications may include early-onset osteoarthritis, extension lag, muscle weakness, recurrence, tibial fractures during casting, valgus deformity post-surgery, and potential future functional impairments [3,7,8].

In the presented case, the newborn also had DDH and generalized ligamentous laxity, as in the literature. The 22q11.22-q11.23 duplication may explain the cardiac, oropharyngeal, and neurodevelopmental anomalies [20]. Despite these comorbidities, conservative treatment of CKD achieved full recovery.

## Conclusions

CKD is a rare congenital disorder requiring prompt diagnosis and early intervention. This case underscores the need for thorough evaluation for associated anomalies and demonstrates the effectiveness of early conservative management. Multidisciplinary follow‑up is critical to ensure favorable long‑term outcomes.
